# New Bioactive Fungal Molecules with High Antioxidant and Antimicrobial Capacity Isolated from *Cerrena unicolor* Idiophasic Cultures

**DOI:** 10.1155/2013/497492

**Published:** 2013-07-15

**Authors:** Magdalena Jaszek, Monika Osińska-Jaroszuk, Grzegorz Janusz, Anna Matuszewska, Dawid Stefaniuk, Justyna Sulej, Jolanta Polak, Marta Ruminowicz, Krzysztof Grzywnowicz, Anna Jarosz-Wilkołazka

**Affiliations:** Department of Biochemistry, Maria Curie-Skłodowska University, Akademicka 19, 20-033 Lublin, Poland

## Abstract

Three bioactive fractions, extracellular laccase (ex-LAC), crude endopolysaccharides (c-EPL), and a low molecular subfraction of secondary metabolites (ex-LMS), were isolated from the idiophasic cultures of the white rot fungus *Cerrena unicolor*. For the first time, we determined the antioxidant properties of these samples by chemiluminometric measurement (a) and assessment of the scavenging effect on ABTS (b) and the DPPH reduction rate (c). The highest reducing capability was found for the ex-LMS fraction: 39–90% for (a), 20–90% for (b), and 10–59% for (c) at the concentration of 6.25–800 *µ*g/mL. The scavenging abilities of the *C. unicolor *c-EPL were between 36 and 70% for (a), 2 and 60% for (b), and 28 and 32% for (c) at the concentration of 6.25–800 *µ*g/mL. A very high prooxidative potential was observed for the ex-LAC probes. The preliminary toxicity tests were done using the Microtox system and revealed the following percentage of the toxic effect against *Vibrio fischeri*: 85.37% for c-EPL, 50.67% for ex-LAC, and 99.8% for ex-LMS, respectively. The ex-LAC sample showed the antibacterial activity against *Escherichia coli*, c-EPL against *Staphylococcus aureus*, and ex-LMS against both bacterial strains, respectively, but the stronger inhibitory effect was exerted on *S. aureus*.

## 1. Introduction

A large number of fungal bioactive compounds, both cellular components and secondary metabolites, have been shown to affect the human immune system and could be used to treat a variety of diseases [1]. Recent intensification in the application of bioactive compounds produced by white rot fungi in the food processing or pharmaceutical industry [2] is stimulating a worldwide search for new natural bioactive compounds of fungal origin. Moreover, their production has become an important field of contemporary biotechnology. A number of these substances including intra- and extracellular low molecular weight compounds, proteins, polysaccharides, or polysaccharide-protein complexes have been isolated from wood-degrading fungal strains [3, 4]. Given the worldwide research, some authors have proposed dividing the bioactive compounds isolated from higher mushrooms into the following groups: (a) secondary metabolites (terpenoids, polyphenols, alkaloids, lactones, sterols, etc.), (b) (glyco)proteins, and (c) high molecular weight polysaccharides [5].

Numerous publications indicate that the most important problem for living organisms is posed by uncontrolled production of reactive oxygen species (ROS), very active by-products having one or more unpaired electrons [6]. Reactive oxygen derivatives are generated during the normal cellular metabolism or as a consequence of exposition to some stress factors, such as changes in temperature, and the presence of metal ions or redox-cycling xenobiotics [6, 7]. As a protection system for the balance between the production and inactivation of ROS, living organisms have evolved specific defense mechanisms for detoxification, consisting of enzymatic and nonenzymatic antioxidants [8]. An uncontrolled increase in the level of free radicals can cause damage to numerous cellular compounds including DNA, proteins, or membrane lipids, in consequence leading to many serious human diseases and disorders, such as atherosclerosis, coronary heart disease, cancer, impaired immune function, or aging processes [6, 9, 10]. This outcome suggests that, with their capacity to scavenge free radicals, antioxidants may protect organisms from oxidative stress-caused damage. Many polysaccharides extracted from *Lentinus polychrous*,* Ganoderma atrum*,* Grifola frondosa*,and *Lentinus edodes *have been reported to have not only antioxidant activity but also they are believed to be bioactive ingredients involved in both the antitumor and anti-inflammatory processes [6, 10]. Furthermore, different other fractions isolated from *Ganoderma lucidum*, *Phellinus linteus*, *Agaricus campestris*, *Lentinus edodes*, *Agaricus blazei,* or *Pleurotus ostreatus *were reported to possess the same therapeutic effects [10–12].

At the same time, investigation and isolation of new natural bioactive substances are also important for food industry due to the growing importance of their antioxidative activity, which is crucial in food preservation processes. Unfortunately, the commonly used synthetic substances such as hydroxyanisole (BHA) and hydroxytoluene (BHT) are likely to be toxic for living organisms [13].

 Interestingly, the physiological life cycle of the white rot Basidiomycota is associated with a relatively high concentration of ROS, which might initiate the secondary wood cell wall decay processes [14]. Therefore, these organisms also possess a very efficient antioxidative system consisting of enzymatic (peroxidases, laccase, catalase, and superoxide dismutase) and nonenzymatic elements (phenolic derivatives or polysaccharides) [15, 16]. Given the information related to the antioxidant potential of wood degrading, medicinal mushrooms [10, 13, 17], in this study we described bioactive compounds derived from the fungus *Cerrena unicolor* belonging to the Polyporaceae family [18], which till now has been poorly examined in this respect. In the present report, the antioxidative properties of crude endopolysaccharides from *C. unicolor *submerged cultures were determined for the first time. It is known that, beside the polysaccharides, fungi are able to produce many secondary metabolites with antioxidative activities including a number of phenolic compounds (e.g., hispidin and its dimmers or fungal pigments usually isolated from fruiting bodies) [13, 19]. Because the white rot fungi are capable of producing large amounts of metabolites to the culture fluid during their growth, we developed a method of isolation of the extracellular low molecular weight subfraction (ex-LMS). This ex-LMS is a side product in the production of biotechnologically important enzymes, for example, laccase. In our work, the antioxidative properties of ex-LMS were tested.

For many years, *C. unicolor* laccase has been studied extensively as a very efficient biocatalyst [20–22]. This multifunction enzyme belongs to the “blue-copper” family of oxidases. A majority of laccases characterized so far have been derived from fungi, especially from white rot Basidiomycotawhich are very efficient lignin degraders [21, 23]. Laccases have also been reported to synthesize products of pharmaceutical importance. Some authors carried out an assay for HIV reverse-transcriptase inhibitory activity using *Tricholoma giganteum *laccase purified from its fruiting body [24]. The laccase inhibited HCV replication and proliferation of hepatoma Hep G2 and breast cancer MCF-7 cells [25]. Although laccase is known to exhibit a significant pharmacological activity, the mechanisms responsible for these properties remain unknown. It is known that the quinone cycle catalyzed by laccase causes oxygen activation, production of superoxide anion radicals, and subsequently production of hydrogen peroxide [16, 26]. It can be assumed that this prooxidative potential of the enzyme is likely to be a tool in their action towards pathogenic cells [25]. This was the reason why the prooxidative properties of *C. unicolor* laccase have been determined in the recent report. 

There are no available reports describing toxicity of extracellular laccase, the crude extract of endopolysaccharides, and the extracellular subfraction of low molecular weight metabolites isolated from the fungus *C. unicolor*. Therefore, the aim of our study, besides the assessment of their anti- or prooxidative potential, was preliminary characterization of these biological samples in terms of their effects on *Vibrio fischeri*, *E. coli*, and *S. aureus* cells.

## 2. Materials and Methods

### 2.1. Strain, Medium, Growth Processing, and Preliminary Separation of Fungal Samples

*C. unicolor *(Bull. ex Fr.) Murr.   was obtained from the culture collection of the Regensburg University and deposited in the fungal collection of the Department of Biochemistry (Maria Curie-Sklodowska University, Poland) under the strain number 139 (ITS sequence deposited in GenBank under accession number DQ056858) [27]. The fermentor scale cultivation was performed at 28°C in a 2.5 L Bioflo III (New Brunswick Scientific, New Brunswick, NJ, USA) fermentor containing 2 L of a sterilized Lindenberg and Holm medium optimized as in [21]. The fermentor was inoculated with crumbled fungal mats (10% of total volume), aerated at 1 L air per minute, and stirred at 100 rpm. Antifoam B emulsion (Sigma, St. Louis, MO, USA) was occasionally added to the fermentor cultures for breaking the foam. The beginning of the idiophase was determined according to Jennings and Lysek's recommendation [28]. 10-day-old idiophasic cultures were filtered through Miracloth (Calbiochem) and used for further assays. After washing with MQ water, the separated fungal biomass was used for isolation of crude endopolysaccharides (c-EPL). The culture liquid obtained after mycelium separation was centrifuged at 10.000 × g for 15 min. The supernatant was subdivided into two fractions on the ultrafiltration system Pellicon 2 Mini holder (Millipore, Bedford, MA, USA) with an Ultracel mini cartridge (10 kD cut-off). The first fraction containing substances above 10 kDa was used as the source of crude laccase, which was purified according to the modified method of Pozdnyakova [29]. The second fraction containing substances below 10 kDa was used as a source of low molecular weight metabolites (extracellular low molecular weight subfraction, ex-LMS).

### 2.2. Preparation of Fungal Bioactive Fractions

#### 2.2.1. Laccase (ex-LAC) Isolation and Detection

The supernatant of the culture fluid was concentrated on the ultrafiltration system Pellicon 2 Mini holder. The concentrated proteins were separated by anion exchange chromatography an a DEAE Sepharose column (2.5 × 15 cm) working under the FPLC system (Bio-Rad, Richmond, VA, USA) equilibrated with 20 mM Tris-HCl buffer (pH 6.5). Proteins were eluted with a linear gradient of NaCl (0.1–0.5 M) at a flow rate of 1 mL/min for 360 min and detected at 280 nm; the fractions containing LAC activity were collected and desalted on the Sephadex G-50 column (5.0 × 20 cm). The purification processes were performed in 4°C. The solution of laccase isoforms mixture was lyophilized using the Freezone 12 Freeze Dry System (Labconco, Kansas City, MO, USA). 

#### 2.2.2. Crude Polysaccharide (c-EPL) Extraction

Mycelia were collected from the cultures by filtration though Miracloth (Calbiochem), washed three times with distilled water, oven-dried at 60°C during 12 hours, and weighed for dry weight. The polysaccharide fractions were extracted from the dried mycelia with hot water (90°C, 4 h) in a 1 : 100 (w/w) ratio, cooled, and then centrifuged at 9.000 × g for 20 min according to modified Freimund's method [30]. The crude polysaccharide was precipitated from the supernatant by four volumes of cold ethanol followed by an overnight incubation at 4°C. The precipitated polysaccharides were collected by centrifugation at 9.000 × g for 20 min and washed three times with ethanol. The samples of the crude polysaccharide were redissolved in distilled water (1 mg/mL) and used for further testing. In the c-EPL obtained, the amount of total and reducing carbohydrates, proteins, and phenolic compounds was examined.

#### 2.2.3. Preparation of the Extracellular Low Molecular Weight Subfraction (ex-LMS)

The subfraction of the culture fluid below 10 kDa, separated by ultrafiltration with a Biomax 10 membrane (10 kDa cut-off), was concentrated using a reverse osmosis column, lyophilized, and used as a source of natural low molecular weight metabolites (extracellular low molecular weight subfraction, ex-LMS). The samples of ex-LMS were dissolved in distilled water (1 mg/mL) and used for further tests. Each sample of ex-LMS was analyzed to assess the amount of total and reducing carbohydrates, proteins, and phenolic compounds.

### 2.3. Analytical Methods

#### 2.3.1. Determination of Carbohydrates, Proteins, and Phenolic Compounds

The total carbohydrate content of c-EPL and ex-LMS was determined by the phenol-sulfuric acid assay with D-glucose as a standard [31]. The concentration of reducing sugars was measured using the Somogyi-Nelson method [32]. The concentration of total polysaccharides was calculated by subtraction of the concentration of reducing sugars separated from the total carbohydrate concentration. Protein concentrations were determined using the Bradford reagent and bovine serum albumin as a standard [33]. The total content of the phenolic compounds was determined with diazosulfanilamide by the DASA test [34], where the absorbance was measured at 500 nm and vanillic acid was used as a standard.

#### 2.3.2. Ex-LAC Activity Assay

LAC activity was measured following oxidation of 0.025 mM of syringaldazine (4-hydroxy 3,5-dimetoxybenzaldehyde) in 50 mM buffer at pH 5.3 [35]. The oxidation of the substrate was recorded at 525 nm at 20°C. One unit of ex-LAC activity was defined as the amount of the enzyme required to oxidize 1 mol of syringaldazine per sec. (the extinction coefficient for production of 65000 M^−1 ^cm^−1^) and expressed in nanokatals per mg of protein (nkat/mg).

### 2.4. Antioxidant Activity Assays

#### 2.4.1. Chemiluminescence (CL) Assay of Antioxidant Activity

Antioxidant properties of the c-EPL, ex-LMS, and ex-LAC fractions were measured using the transient property of the Fenton reaction and scavenging reaction between luminol and hydroxyl radicals described by Cheng et al. [36]. The reaction mixture contained 1.5 mM Fe^2+^-EDTA solution, 4.4 mM H_2_O_2_ and 2 mM luminol in 95% ethanol, 50 mM phosphate buffer (pH 7.4), and the tested samples. The chemiluminescent (CL) reaction was conducted at room temperature. The samples of the tested compounds (100 *μ*L) at concentrations ranging from 6.25 to 800 *μ*g/mL were added to 600 *μ*L buffer and 100 *μ*L of luminol and mixed. In the next stage, 100 *μ*L of Fe (II) and 100 *μ*L of H_2_O_2_ were added to the reaction mixture. The Fe (II)-H_2_O_2_-luminol reaction signal profiles were detected by a Lumat LB 9506 luminometer (Berthold, Germany) with two dispensers. The CL peak values were recorded in the absence (*I*_0_) or presence (*I*_1_) of the tested compounds. The inhibitory rate (*I*_*R*_) was calculated according to the following equation:

(1) $$\begin{array}{c} I_{R}=\left(1-\frac{I_{1}}{I_{0}}\right)\times 100\%. \end{array}$$

The trolox calibration curve was prepared in the range of the concentrations from 6.25 to 800 *μ*g/mL and EC_50_ values were obtained. EC_50_ value is described as the effective concentration at which the radicals present in the investigated samples were scavenged by 50%; the antioxidant activity was 50%.

#### 2.4.2. ABTS Radical-Scavenging Test

The ABTS radical-scavenging activities of the c-EPL, ex-LMS, and ex-LAC fractions were conducted according to the method of van den Berg et al. [37], Duo-Chuan [38], and Re et al. [39] with modification. The stock solution was prepared by dissolving 7.4 mM ABTS and 2.6 mM potassium persulfate in MQ water, and this solution was stored in the dark for 16 h at room temperature before use. In the next stage, the concentrated ABTS stock solution was diluted with phosphate buffered saline (PBS) pH 7.4 to absorbance 0.700 ± 0.02 recorded at 734 nm. For measuring the antioxidant capacity, 10 *μ*L of the tested compound at concentrations ranging from 6.25 to 800 *μ*g/mL was mixed with 990 *μ*L of the ABTS radical solution. The percentage of inhibition of ABTS oxidation was calculated by the following formula:

(2) $$\begin{array}{c} {\text{ABTS}}^{∙+}\;\;\text{scavenging}\;\;\text{effect}\;\;\left(\%\right)=\left[\frac{\left(A_{0}-A_{1}\right)}{A_{0}}\right]\times 100, \end{array}$$

where *A*_0_ means the absorbance of the control and *A*_1_ the absorbance at 734 nm of the tested compound/standard. The trolox calibration curve was prepared for a concentration range from 6.25 to 800 *μ*g/mL and EC_50_ values were obtained.

#### 2.4.3. DPPH Free Radical-Scavenging Test

The DPPH free radical-scavenging activity of the c-EPL, ex-LMS, and ex-LAC fractions was estimated according to the procedure described by Paduch et al. [40]. This method is based on the ability of 1,1-diphenyl-2-picrylhydrazyl (DPPH), a stable free radical, to decolorize in the presence of antioxidants. The tested compound (0.1 mL) at concentrations ranging from 6.25 to 800 *μ*g/mL was added to 0.1 mL of a DPPH^*∙*^ solution (0.2 mg/mL in ethanol). Trolox and ascorbic acid (Vit. C), the well-known standards with strong antioxidant activities, were used as positive controls. Absorbance at 515 nm was determined after 2, 5, 10, 15, 20, and 30 min of incubation at room temperature. The percentage of inhibition of DPPH oxidation was calculated according to the following formula:

(3) $$\begin{array}{c} {\text{DPPH}}^{∙}\;\;\text{scavenging}\;\;\text{effect}\;\;\left(\%\right)=\left[\frac{\left(A_{0}-A_{1}\right)}{A_{0}}\right]\times 100, \end{array}$$

where *A*_0_ means the absorbance of the control sample and *A*_1_ means the absorbance of the standard or tested compound. The inhibition curves were prepared and EC_50_ values were obtained.

### 2.5. FT-IR Spectroscopy Analysis of c-EPL and ex-LMS Samples

To determine the composition of the endopolysaccharides (c-EPL), their completed acid hydrolysis was carried out with 4.95 N trifluoroacetic acid (TFA) at 80°C in a heating block for 4 h. The mixture was cooled to the room temperature, evaporated, and then analyzed by infrared spectroscopy. The analyses of ex-LMS were performed using the crude lyophilized filtrate obtained from the concentrated culture fluid separated with a Biomax 10 kDa membrane. FT-IR spectroscopy was recorded with a spectrometer (Thermo Scientific Nicolet 8700A with FT Ramana Nicolet NXR module) in the wavelength range 4000–400 cm^−1^.

### 2.6. Visualization of Endopolysaccharides Using Confocal Laser Scanning Microscopy

Fluorescence Brightener 28 was used in order to detect *β*-linked polysaccharides. The lyophilized samples of c-EPL (1 mg) were washed one time with MQ water and, after centrifugation at 10000 ×g for 5 min, stained for 30 min with 200 *μ*L of 25 *μ*g/mL Fluorescence Brightener 28. In the next step, the dye solution was removed and the precipitate was washed two times with MQ water, placed on a glass slide, and observed under a microscope. For visualization of the endopolysaccharides, an inverted microscope Axiovert 200 M equipped with an LSM 5 Pascal head (with magnification 200x) was used.

### 2.7. Estimation of the Toxicity Effect of the c-EPL, ex-LMS, and ex-LAC Fractions

The toxic effects of the tested fractions from *C. unicolor *cultures against marine bacterium *Vibrio fischeri* were estimated using the Microtox Model 500 Analyzer detection system. The test is based on the luminescence ability of the bacteria. Any inhibition of cell respiration is correlated with the cellular activity and results in a reduction of luminescence. The light output of *V*.* fischeri* was measured at 0, 5, and 15 min after the treatment with c-EPL, ex-LMS, and ex-LAC. The analytical procedure was applied according to the Screening Test Protocol of the Microtox assay.

### 2.8. Analysis of the Antibacterial Activity of the ex-LAC, c-EPL, and ex-LMS Fractions

The antibacterial activity of the samples was studied by the method of well diffusion assay with slight modifications. *Escherichia coli *(ATCC 25992) and *Staphylococcus aureus* (ATCC 25923) were used as indicator bacteria and inoculated into commercially available Muller-Hinton Agar II medium (LabM (TM), IDG plc, UK), 38 g/L, with the inoculum solution (100 *μ*L) about 1 × 10^5^ CFU/mL of each kind of microorganism smeared on the standard assay medium. Volume of 100 *μ*L of the specimen (concentration 1 mg/mL) was added into the agar well in the center of the Petri dishes and left to incubate for 2 hours at room temperature; afterwards the plates were transferred to the 37°C for 18 h. Sterilized physiological saline was used as a control. After the medium was incubated, the inhibition zones were measured.

### 2.9. Statistical Analysis

All the results are expressed as mean ± SD from three experiments (*n* = 3). Values of *P* ≤ 0.05 were only reported as a statistically significant. The mean values and standard deviation were calculated using the Excel program from Microsoft Office 2010 package.

## 3. Results and Discussion

For the past two decades, *C. unicolor* regarded as a nonedible fungus has been intensively studied as a very efficient source of extracellular laccase produced in noninduced conditions of growth [20, 21, 41]. *C. unicolor* belongs to the phylum Basidiomycota consisting of a number of species that are the most versatile among other wood-decaying species and are very useful biotechnological tools in various industrial processes [13]. Much attention has been directed especially to the typical edible mushrooms belonging to Basidiomycota, which are being evaluated for their nutritional and pharmacological properties. On the other hand, many untypical edible species of this phylum are completely unknown as a source of pharmaceutical and nutritional substances.

In the present work, it was detected that the 10-day-old idiophasic cultures of *C. unicolor *could be used as a source of three fractions of potentially bioactive fungal metabolites: extracellular laccase (ex-LAC), endopolysaccharides (c-EPL), and the extracellular subfraction of low molecular weight compounds (ex-LMS). According to the available knowledge, this is the first attempt at testing this type of preparations from *C. unicolor* in terms of their antioxidant properties and toxicity.

### 3.1. Determination of Extracellular Laccase (ex-LAC) Properties

#### 3.1.1. General

The extracellular laccase was isolated and partially purified from the idiophasic cultures of *C. unicolor *according to Pozdnyakova et al. [29]. 1 mg of lyophilized laccase isoforms mixture dissolved in 1 mL of MQ water possessed the activity of 1 150 110 nkat and protein concentration c = 329 *μ*g/mL. The specific activity of the laccase preparation was 3495.4 nkat/mg of protein. It has been reported that *C. unicolor* is an excellent source of very active ex-LAC [20, 21, 41]. In contrast to the many laccase producers like *T. versicolor*, this fungus is able to produce high amounts of this enzyme without any extra supplementation, for example, some aromatic compounds [41, 42]. This outcome may be exploited to produce high amounts of this enzyme as a biologically active substance with pharmacological potential.

#### 3.1.2. Prooxidative, Toxic, and Antibacterial Effects

**Figure 1:**
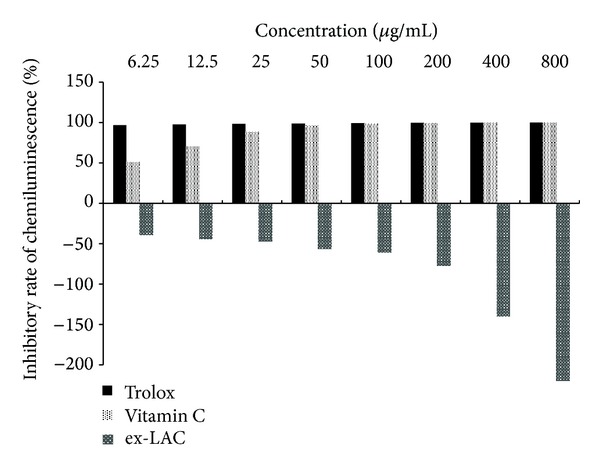
Inhibitory rate of laccase (ex-LAC) from *C. unicolor *assessed with the chemiluminescence method. Data are mean ±  SD for three measurements (*n* = 3).

Some available reports proposed using LAC from other fungi (*Clitocybe maxima*, *P. ostreatus,* or *P. eryngii*) as cytotoxic and antiviral agents [24, 42–45], but the mechanisms of this action are still unknown [25]. The prooxidative properties of ex-LAC have been described in relation to quinone cycles catalyzed by an enzyme causing oxygen activation, production of superoxide anion radicals, and the subsequent production of hydrogen peroxide [16, 26]. Based on these data, we can speculate that one possibility of damage mechanisms towards pathogenic cells can be probably based on the prooxidative action of this enzyme, especially in the presence of redox cycling compounds. There are no data describing this white rot fungus enzyme from this point of view. Having measured the potential of the investigated ex-LAC to produce reactive oxygen species (ROS) using chemiluminometric detection, a very strong prooxidative action of the investigated enzyme was found (Figure 1). 200% higher levels of ROS production were observed in the tested samples for amounts of proteins corresponding to the 800 *μ*g/mL of trolox and ascorbic acid used as controls. Luminol is widely used for studying radical reactions and is accepted when a single oxidant, likewise the purified ex-LAC used in the present paper, is measured [46]. The obtained results showed linearity dependence between the chemiluminescence and enzyme concentration. Because the compounds of the reaction mixture used in ABTS and DPPH free radical scavenging test inhibited the ex-LAC activity, they are not proper for this kind of estimation (data not shown). The estimation of ex-LAC toxicity using a Microtox detection system showed that the exposure of the marine bacterium V*ibrio fischeri* to ex-LAC caused 38% cells damage after 5 min and 51% after 15 min treatment with this enzyme. The ex-LAC sample was found to be effective against *E. coli*, with the inhibition zone of 13.66 mm (Table 3). The preliminary results obtained are in agreement with the idea of the antimicrobial, antiviral, and antiproliferative actions of selected fungal proteins, including laccase [1].

### 3.2. Properties of the Crude Extract of Endopolysaccharides (c-EPL)

#### 3.2.1. General

**Figure 2:**
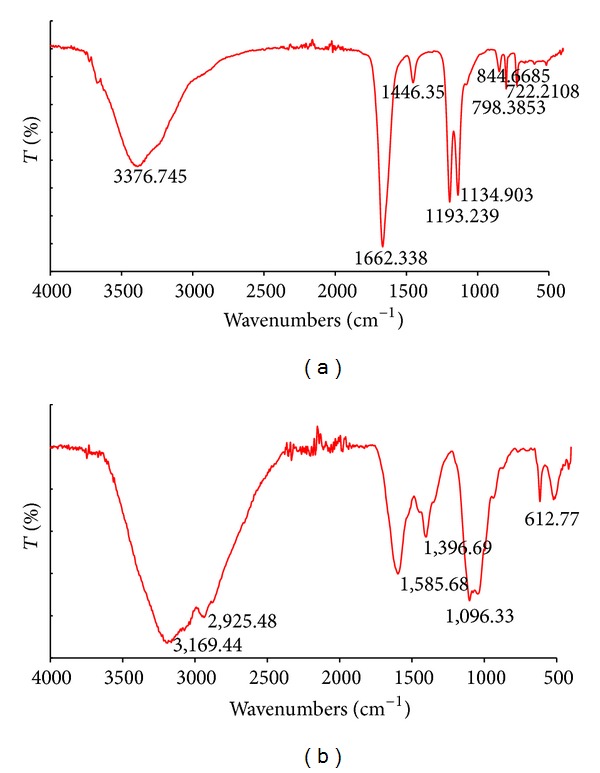
FT-IR spectra of the crude extract of hydrolyzed endopolysaccharides: (a) c-EPL and extracellular subfraction of low molecular weight metabolites and (b) ex-LMS from *C. unicolor. *

Recently, fungal polysaccharides have been proposed as a very promising factor for various industrial applications including biopharmacy and cosmetology. The most abundant fungal mushroom polysaccharides are chitin, *α*- and *β*-glucans, xylans, mannans, and galactans [47]. In the present report, the FT-IR spectra of the crude endopolysaccharides (c-EPL) isolated from *C. unicolor* showed a typical carbohydrate pattern. As shown in Figure 2(a), the FT-IR spectrum of c-EPL displays a broad stretching intense characteristic peak at 3376 cm^−1^ characteristic for the hydroxyl group [11]. The absorption bands at 1662 and 1446 cm^−1^ suggest the presence of the deprotonated carboxylic group (COO^−^) and proteins which were detected in crude polysaccharide extract [48, 49]. The sharp bands at 1193 and 1134 cm^−1^ in the FT-IR spectra indicate the presence of C–O bonds. Characteristic *α*-linked glycosyl residues in the polysaccharides at 844 cm^−1^ were also present [50]. The determination of the chemical properties of c-EPL showed the total carbohydrate content reaching 8.77% of the extract (Table 1). The available data of *Lentinus edodes* show that crude endopolysaccharides isolated from mycelia contained 22.8% of total carbohydrates. The values obtained for the samples extracted from the fresh fruiting bodies of *Lentinus polychrous *were significantly higher (45.9%) in comparison to the mycelia obtained from submerged cultures [10]. The total polysaccharides of the presented c-EPL (73.2 mg/g of dry mycelia/732.5 *μ*g/mL of sample solution) (Table 1) were higher than those reported for *Ganoderma lucidum* (27.6 mg/g) or *Agaricus brasiliensis* (45.9 mg/g) and on a similar level as for *Agaricus bisporus* (74.4 mg/g) [11]. The level of reducing sugars was lower than that in the described strains and reached 14.5 mg/g of dry mycelia (145 *μ*g/mL of sample solution) (Table 1). The concentration of phenolic compounds in the extracted c-EPL was 77 *μ*M. The total phenol content in the case of *Lentinus polychrous *mycelia was 58.4 mg/g [10]. 270 *μ*g/mL of proteins was detected in the c-EPL extracted from *C. unicolor *mycelium. Staining *of *c-EPL preparation with Fluorescence Brightener 28 (binding to *β*-linked polysaccharides) confirmed the presence of sugars in this sample (Figure 6). The comparison of the FT-IR spectrum for c-EPL with the library data indicates the presence of substances with 46.68% homology to dihydroxystreptomycin, a derivative of aminoglycoside antibiotics.

#### 3.2.2. Antioxidative Properties

**Figure 3:**
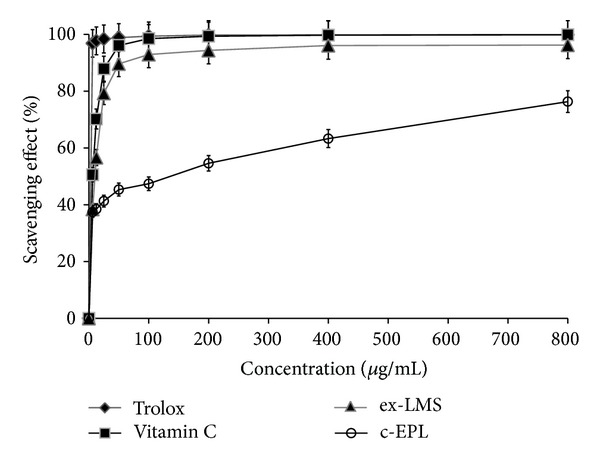
Inhibitory rate of the crude extract of endopolysaccharides (c-EPL) and extracellular sub-fraction of low molecular weight metabolites (ex-LMS) from *C. unicolor *assessed with the chemiluminescence method. Data are mean ±  SD for three measurements (*n* = 3).

**Figure 4:**
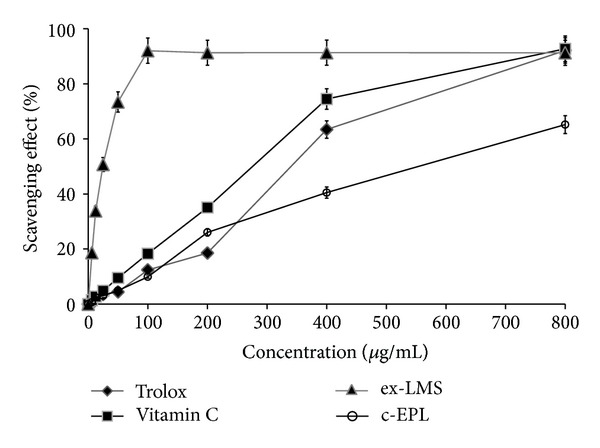
Scavenging effects of the crude extract of endopolysaccharides (c-EPL) and extracellular sub-fraction of low molecular weight metabolites (ex-LMS) from *C. unicolor *assessed with the ABTS radical-scavenging method. Data are mean  ±  SD for three measurements (*n* = 3).

**Figure 5:**
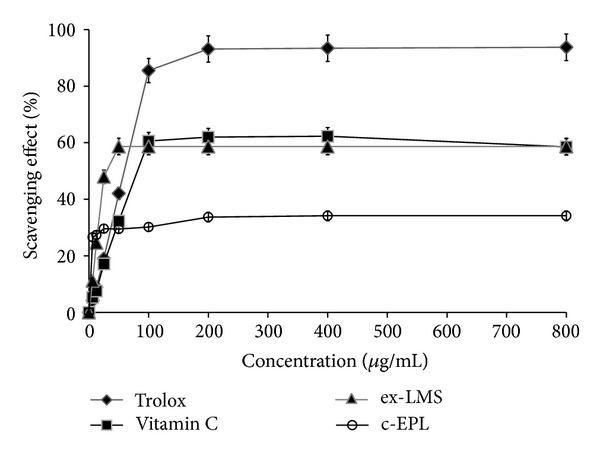
Scavenging effects of the crude extract of endopolysaccharides (c-EPL) and extracellular sub-fraction of low molecular weight metabolites (ex-LMS) from *C. unicolor* assessed with the DPPH radical-scavenging method. Data are mean ± SD for three measurements (*n* = 3).

**Figure 6:**
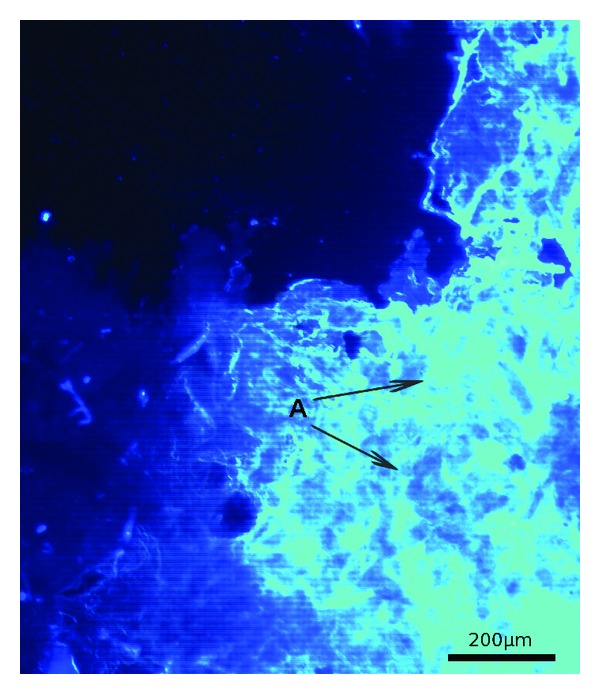
Visualization of endopolysaccharides using confocal laser scanning microscopy. The lyophilized samples of c-EPL, washed with MQ water, were stained for 30 min with 200 *μ*L of 25 *μ*g/mL Fluorescence Brightener 28 commonly used in order to detect *β*-linked polysaccharides. For visualization of the endopolysaccharides from *C. unicolor,* the inverted microscope Axiovert 200 M equipped with an LSM 5 Pascal head (with magnification 200x) was used. The letter (A) indicates the luminous areas exhibiting visible *β*-linked polysaccharide fragments.

Polysaccharides from mushrooms have often been described as anticancer, antiviral, anticoagulating, and immunostimulating compounds [10, 11]. In many papers, polysaccharides isolated from strains for example, *Ganoderma atrum*, *Lentinus edodes*, *Agaricus bisporus*, *Phellinus linteus*,and* Lentinus polychrous *[10–12], were tested as antioxidative factors. The antioxidative properties of *C. unicolor *c-EPL were tested using the chemiluminescence method with luminol and spectrophotometric free radical-scavenging tests with ABTS and DPPH used as substrates. The reducing properties of extracted c-EPL were evaluated in trolox and ascorbic acid (Vit. C) model systems. At the concentration range of 6.25–800 *μ*g/mL, the scavenging abilities of* C. unicolor *c-EPL were 36–70% for luminol, 2–60% for ABTS, and 28–32% for DPPH (Figures 3, 4, and 5). It was described that 5 mg/mL of hot water extracted *Ganoderma tsugae *endopolysaccharides exhibited the scavenging ability at the 36.4–58.4% level in the DPPH test [51].

The calculated EC_50_ values reflected the concentration of the tested compounds able to scavenge 50% of free radicals present in the reaction mixture (Table 2). The lowest values of EC_50_ were observed for the chemiluminometric method, that is, 183.15 *μ*g/mL for c-EPL, 0.39 *μ*g/mL for trolox, and 6.25 *μ*g/mL for ascorbic acid. The EC_50_ values for the ABTS- and DPPH-scavenging methods used for testing the c-EPL antioxidative potential amounted to 493.8 *μ*g/mL and >800 *μ*g/mL, respectively. All these data indicate that c-EPL extracted from *C. unicolor *possesses a high antioxidative potential comparable to the reported hot water extracted fungal polysaccharides, *Agaricus brasiliensis, Ganoderma lucidum, Ganoderma applanatum, Lentinus edodes, *and* Trametes versicolor *[11, 49]. The threefold estimation evaluated in our paper showed that the best antioxidative capacity was detected using chemiluminometric measurements. This dependence may be associated with the fact that CL is a very effective method for evaluation of phenolic antioxidants which are present also in the c-EPL from *C. unicolor *[36]. To sum up, the high antioxidative capacity of the c–EPL from *C. unicolor *was confirmed and it was higher than the antioxidative properties of polysaccharides isolated from other fungi, for example, *Agaricus bisporus* (obtained from hot water extracted fresh fruit bodies) [11] or *Agaricus blazei* and *Lentinus edodes *(obtained from dry powder formulation) [52]. In the case of polysaccharides isolated from these strains, the EC_50_ values (measured with DPPH) were on the levels 2.0 mg/mL, 6.77 mg/mL, and 26.32 mg/mL, respectively [11, 52]. Considering these facts, *C. unicolor* mycelium seems to be a very promising new source of endopolysaccharides with important antioxidant properties.

#### 3.2.3. Toxic and Antimicrobial Effect

Extracts of polysaccharides isolated from mushrooms have been reported to stimulate or suppress specific components of the immune system and can be a useful adjunct to conventional therapy for cancer or the other diseases [53]. Preliminary assessment of the c-EPL from *C. unicolor *as an anti-inflammatory agent based on the bacterial toxicity test using the Microtox detection system showed that the exposure of marine bacterium *Vibrio fischeri* to c-EPL caused 85% cell damage after 5 min and 88% after 15 min of polysaccharide treatment. *E. coli* and *S. aureus* were used to study the antibacterial activity of the isolated fractions. The c-EPL sample showed the antibacterial activity against *S. aureus *with the inhibition zone of 18.96 mm (Table 3). The preliminary results obtained suggest that the investigated sample of c-EPL may be a possible source of natural bacteriostatic agents.

### 3.3. Properties of the Extracellular Low Molecular Fraction (ex-LMS)

#### 3.3.1. General

The studies showed the possibility of a new way of using the common waste product formed during the production of extracellular enzymes by *C. unicolor* as a source of low molecular antioxidants, simple sugars, or active peptides. The analysis of the chemical composition of ex-LMS showed the presence of sugars (total carbohydrate: 780.07 *μ*g/mL, reducing sugars: 507.14 *μ*g/mL, total polysaccharides: 272.93 *μ*g/mL, proteins (189 *μ*g/mL), and phenolic compounds (15 *μ*M) (Table 1)). The FT-IR spectrum analysis of the low molecular weight subfraction of metabolites (ex-LMS) of the *C. unicolor* idiophasic cultures demonstrated an aminoglycoside substance pattern (Figure 2(b)). The characteristic strong broad band at 3169 cm^−1^ indicates the presence of OH stretching in hydrogen bonds [11]. The absorption bands at 1585 and 1396 cm^−1^ are attributed to the stretching vibration of the C–O bond of the carboxyl group, characteristic for proteins [48]. The band between 1000 and 1100 cm^−1^ (i.e., 1096 cm^−1^) indicates the presence of O-substituted glucose residues and *β*-linkages in the glucosidic chain [54]. The absorption band at 612 cm^−1^ suggests that the ex-LMS fraction contains pyranose rings in its structure. Additionally, the comparison of the results from the library indicates the presence of substances homologous in 54.22% to paromomycin sulfate, a compound that is a derivative of the aminoglycoside antibiotics in the ex-LMS isolated from *C. unicolor*.

#### 3.3.2. Antioxidative Properties

Mushrooms have often been described as very efficient producers of extracellular secondary metabolites in the idiophasic cultures. Some authors have described isolation of polyphenols with antioxidative properties from the culture broth of *Inonotus xeranticus* and *Phellinus linteus *[13]. The investigation of ex-LMS obtained from *C. unicolor* culture fluid after separation of compounds above 10 kDa showed a very strong reducing activity of this subfraction. The measurements performed with three methods (chemiluminescence of luminol and ABTS and DPPH radical-scavenging tests) showed the scavenging ability of ex-LMS on the same level or even higher in comparison to the model antioxidative substances, trolox and ascorbic acid. The scavenging abilities of *C. unicolor* ex-LMS at the concentration range of 6.25–800 *μ*g/mL were between 39 and 90% for luminol, 20 and 90% for ABTS, and 10 and 59% for DPPH (Figures 3–5). According to our knowledge, there are no adequate data comparable to the data obtained in the present paper. The ex-LMS fraction is a mixture composed of different low molecular substances that may interact with each other and act together. The analytical measurements showed the presence of sugars, proteins, and phenolic compounds in this fraction. Some data showed that the extracellular culture fluid of *Pycnoporus sanguineus* cultures is a source of antibacterial substances [55]. Other biologically active substances such as vitamins A, B1, B2, B12, and C or bioactive peptides are also the products of fungal fermentations [1, 17].

The calculation of the EC_50_ normalized values described the concentration of fungal samples that are able to scavenge 50% of free radicals present in the reaction mixture (Table 2). The lowest values of EC_50_ detected for the chemiluminometric method amounted to 11.04 *μ*g/mL for ex-LMS, 0.39 *μ*g/mL for trolox, and 6.25 *μ*g/mL for ascorbic acid. EC_50_ values in the case of the ABTS- and DPPH-scavenging methods were 25.0 *μ*g/mL and 85.3 *μ*g/mL, respectively. This very effective scavenging ability of the ex-LMS from *C. unicolor *culturesmay highlight this subfraction as a very interesting source of easily isolated natural antioxidants. Our initial experiments proved that the chemiluminometric method seems to be the best for estimation of antioxidative capacity in the case of ex-LMS. It is very interesting that this fraction, very rich in reducing sugars, is more effective in ROS scavenging than the c-EPL isolated from the mycelium of *C. unicolor.*

#### 3.3.3. Toxic and Antibacterial Effects

The investigation of the toxic effect of theex-LMS from *C. unicolor *based on the bacterial toxicity test using the Microtox detection system showed that the exposure of the marine bacterium V*ibrio fischeri* to the low molecular weight subfraction of extracellular metabolites caused 95% cell damage after 5 min and 98% after 15 min of sample addition. The results obtained showed that the ex-LMS was much more effective than the ex-LAC fraction and similar to the c-EPL in the action on the bacterial cells. *E. coli* and *S. aureus* were used to study the antibacterial activity of the isolated fraction. The ex-LMS sample showed the antibacterial activity against both *E. coli *and* S. aureus,* respectively, with the inhibition zone of 11.83 mm (*E. coli*) and 25.86 mm (*S. aureus*) (Table 3). This may be connected with the fact that the FT-IR analysis has indicated the presence of derivatives, similar to known aminoglycoside antibiotics, in the investigated samples (ex-LMS, c-EPL).

## 4. Conclusion

Our paper proposes a new insight into the possibility of application of common wood-destroying fungus *C. unicolor* as a source of three fractions of potentially bioactive metabolites: extracellular laccase (ex-LAC), intracellular nonpurified polysaccharides (c-EPL), and a low molecular weight subfraction of extracellular metabolites (ex-LMS). Each of them can be investigated differentially. 

To the best of our knowledge, this is the first report describing the very high ROS-scavenging potential of fungal preparations such as endopolysaccharides and extracellular low molecular weight compounds measured by three different methods. These substances may potentially be used as a new source of effective antioxidants that can be easily produced in controlled laboratory conditions. Therefore, the results obtained introduce a new, nonedible fungus to the medicinal mushroom family, comprising species like *Trametes versicolor* (crestin source) and *Schizophyllum commune* (schizophyllan source), which are regarded as nonedible strains but as producers of bioactive substances.

The prooxidative potential of laccase and the toxic effect on bacterial cells of all the three fractions suggest continuation of the presented studies in terms of pharmacological effects. However, it is worth noting that further studies are needed comprising isolation and characterization of the above-mentioned bioactive substances, and their possible use as crucial factors in new therapy and as a natural source of antioxidative molecules.

## Figures and Tables

**Table 1:** Chemical composition of *C. unicolor* c-EPL and ex-LMS: yield of total carbohydrate, reducing sugars, concentration of phenolic compounds, proteins contents, and total polysaccharides.

Samples	Protein (*μ*g/mL)	Total carbohydrate (*μ*g/mL)	Total phenolic (*μ*M)	Reducing sugars (*μ*g/mL)	Total polysaccharides (*μ*g/mL)
ex-LMS	188.97 ± 1.3^a^	780.07 ± 2.7^a^	15.0 ± 0.4^a^	507.14 ± 2.8^a^	272.93 ± 2.7^a^
c-EPL	270.0 ± 2.6^b^	877.3 ± 2.1^b^	77.0 ± 1.3^b^	144.8 ± 1.5^b^	732.5 ± 2.5^b^

All results are expressed as mean ± SD from three experiments (*n* = 3). Values with different letters within the columns are significantly different (*P* ≤ 0.05).

**Table 2:** EC_50 _values (effective concentration at which the radicals present in the investigated samples were scavenged by 50%; the antioxidant activity was 50%) of c-EPL and ex-LMS isolated from *C. unicolor* submerged cultures in comparison to trolox and Vit C.

EC_50 _(*μ*g/mL)				
	c-EPL	ex-LMS	trolox	Vit C
chemiluminescence method	183.15 ± 1.2	11.04 ± 0.2	0.39 ± 0.1	6.25 ± 0.2
ABTS radical scavenging	493.8 ± 2.2	25.0 ± 0.1	315.9 ± 2.1	268.4 ± 2.2
DPPH radical scavenging	>800	85.3 ± 0.7	59.52 ± 0.7	82.56 ± 1.1

All results are expressed as mean ± SD from three experiments (*n* = 3). Values within the column and the row for investigated samples are significantly different (*P* ≤ 0.05). EC_50_ > 800 *μ*g/mL cannot be calculated from the graphs.

**Table 3:** The antibacterial activities of ex-LAC, c-EPL, and ex-LMS (1 mg/mL) isolated from *C. unicolor* submerged cultures.

	Diameters of inhibition zone (mm)	
	*E. coli *	*S. aureus *
ex-LAC	13.66 ± 0.4	—^a^
c-EPL	—	18.96 ± 0.4
ex-LMS	11.83 ± 0.2	25.86 ± 0.2
Physiological saline	—	—

All results are expressed as mean ± SD from three experiments (*n* = 3). Values within the columns are significantly different (*P* ≤ 0.05). ^a^Not detected.
